# The pharmacokinetic challenge of treating invasive aspergillosis complicating severe influenzae assisted by extracorporeal membrane oxygenation

**DOI:** 10.1186/s13054-018-2285-5

**Published:** 2018-12-22

**Authors:** Hadrien Winiszewski, Anne-Claire Rougny, Jennifer Lagoutte-Renosi, Laurence Millon, Gilles Capellier, Jean-Christophe Navellou, Gael Piton, Anne-Laure Clairet

**Affiliations:** 1 Medical Intensive Care Unit, Besançon, France; 2 Department of Pharmacy, Besançon, France; 3 Department of Clinical Pharmacology, Besançon, France; 4 Department of Clinical Mycology, Besançon, France; 5 Department of Epidemiology and Preventive Medicine, School of Public Health and Preventive Medicine, Faculty of Medicine, Nursing and Health Sciences, Clayton, Australia; 6 Research Unit EA 3920 and SFR FED 4234, University of Franche Comté, Besançon, France

Invasive aspergillosis (IA) complicates 19% of patients requiring ICU admission for severe influenza [1]. Despite recent development of isavuconazole, voriconazole remains the first therapeutic option for IA among patients without hematological malignancy [2]. However, among patients requiring the use of extracorporeal membrane oxygenation (ECMO) as rescue therapy for extremely severe acute respiratory failure [1], the use of ECMO per se has been reported as a cause of sequestration of voriconazole on the membrane. This phenomenon could induce low plasma concentrations of voriconazole and, therefore, limited efficacy [3].

A 57-year-old man with no significant medical history was admitted to our ICU for severe influenza. After intubation, broncho-alveolar lavage revealed the presence of *Aspergillus fumigatus*. Galactomannan antigen and PCR for *Aspergillus* DNA were also positive in epithelial lining fluid. A computed tomography scan was consistent with the diagnosis of IA and intravenous voriconazole was started with usual doses. After one week, despite maximal respiratory management, veno-venous ECMO (CardioHelp MAQUET system, Getinge society) was introduced because of refractory respiratory acidosis. Therapeutic drug monitoring was performed targeting a residual concentration between 2 and 6 mg/L. Weaning of ECMO was not possible and the patient stayed under ECMO support for 4 months. As a consequence, a total of five ECMO membranes were needed. We observed after every membrane change a drop of voriconazole plasma concentration, with the need to increase the dose (Fig. 1). Because of the high pharmacokinetic variability, a combination therapy of voriconazole with liposomal amphotericin B was started from week 2. Despite such combination therapy and therapeutic drug monitoring, failure to wean the patient from ECMO led to care withdrawal after 5 months.

**Fig. 1:**
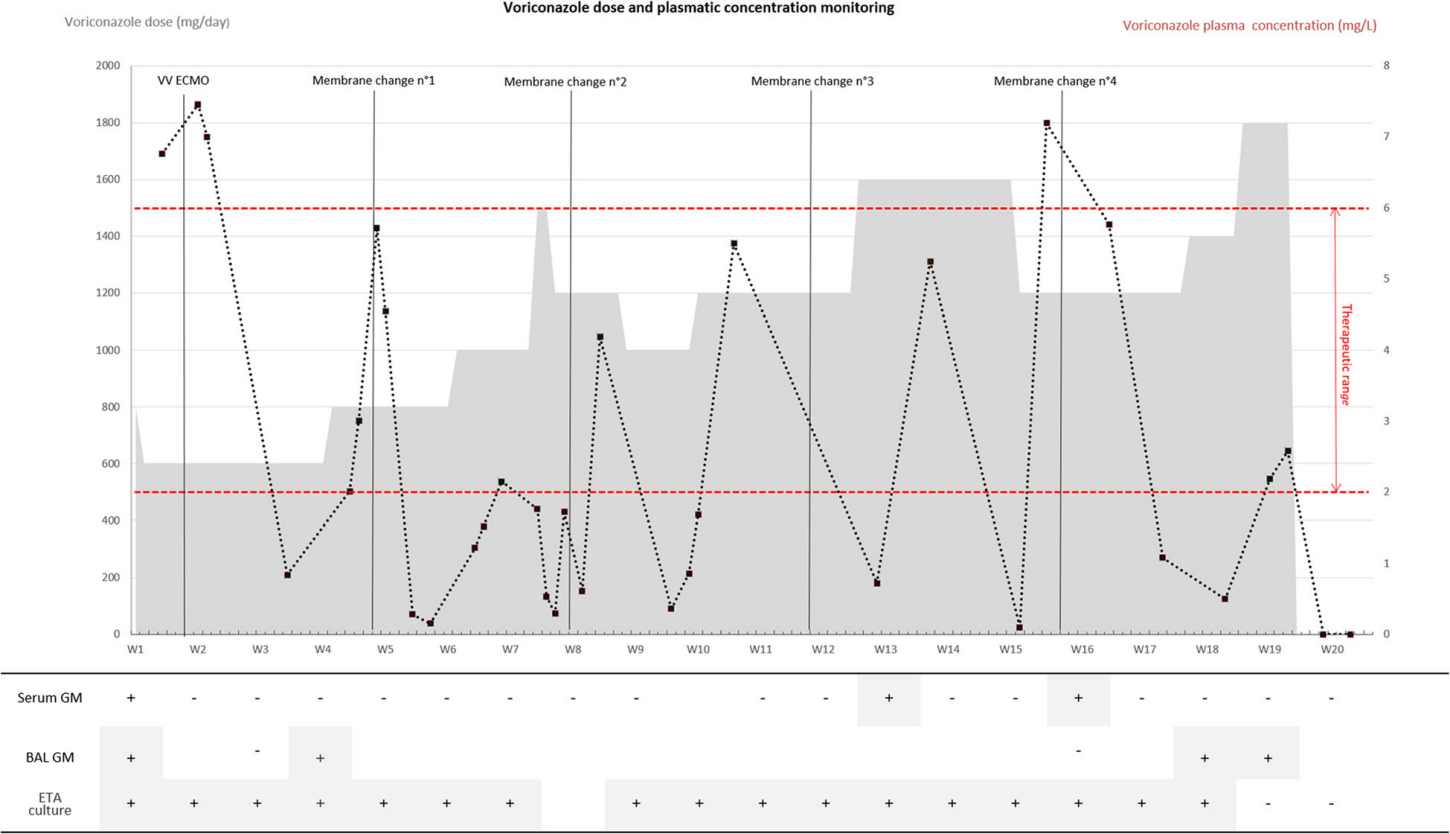
Evolution of voriconazole dose and plasma residual concentration during the ICU stay. The x-axis corresponds to time (weeks). The left y-axis corresponds to the voriconazole dose (mg/day). The *gray shaded area* corresponds to the dose evolution of voriconazole. The right y-axis corresponds to the voriconazole plasma concentration (mg/L). *Black dotted line* corresponds to evolution of voriconazole plasma concentration. *Upper* and *lower red dotted line* correspond to the therapeutic range of voriconazole plasma concentration (i.e., 2–6 mg/L). The lower part of the figure corresponds to evolution of serum galactomannan antigen (*serum GM*), broncho-alveolar lavage galactomannan antigen (*BAL GM*), and endo-tracheal aspirates culture for aspergillus (*ETA culture*)

In the present case, the diagnosis of refractory IA was considered since lung infiltrates and endo-tracheal aspirate cultures of *Aspergillus fumigatus* persisted during the entire ICU stay. In addition, galactomannan antigen was still detected in epithelial lining fluids after 18 weeks of treatment by voriconazole. Such treatment failure was probably linked to frequent voriconazole underdosing. Indeed, 17 out of the 32 times therapeutic drug was monitored showed voriconazole concentrations under the target of 2 mg/L despite high dose administration (until 12 mg/kg twice a day).

Sequestration of voriconazole, a lipophilic drug, on the ECMO membrane is a well-known phenomenon [3]. As no drug–drug interaction was detected in the present case, sequestration of voriconazole was likely the main reason leading to such pharmacokinetic variability. After 2 or 3 weeks of membrane use, the membrane was well saturated by voriconazole, resulting in sufficient and stable plasma concentrations in the patient. However, immediately after the membrane change, intense adsorption of voriconazole on the new membrane was likely responsible for the drop in plasma concentration. Several days of high voriconazole dosing were then required before the plasma concentration reached the target concentration.

Given the high number of IA complicating influenza, and the increasing development of the ECMO technique for refractory acute respiratory failure, the described situation might become frequent. Data regarding the pharmacokinetics and efficacy of other antifungal drugs, such as isavuconazole, during ECMO therapy are urgently needed.

## Abbreviations

CT: Computed tomography
ECMO: Extracorporeal membrane oxygenation
IA: Invasive aspergillosis
ICU: Intensive care unit
PCR: Polymerase chain reaction
